# Combined Pre- and Postnatal Minimally Invasive Approach to a Complex Symptomatic Congenital Pulmonary Airway Malformation

**DOI:** 10.1055/a-2107-0409

**Published:** 2023-07-17

**Authors:** Francesco Macchini, Stefano Mazzoleni, Giacomo Cavallaro, Nicola Persico, Irene Borzani, Ernesto Leva

**Affiliations:** 1 Department of Pediatric Surgery, Fondazione IRCCS Ca' Granda Ospedale Maggiore Policlinico, Milan, Lombardia, Italy; 2 Department of Neonatal Intensive Care Unit, Fondazione IRCCS Ca' Granda Ospedale Maggiore Policlinico, Milan, Lombardia, Italy; 3 Department of Obstetrics and Gynecology, Fondazione IRCCS Ca' Granda Ospedale Maggiore Policlinico, Milan, Lombardia, Italy; 4 Department of Pediatric Radiology Unit, Fondazione IRCCS Ca' Granda Ospedale Maggiore Policlinico, Milan, Lombardia, Italy

**Keywords:** congenital pulmonary airway malformation, thoraco-amniotic shunt, thoracoscopic lobectomy, neonate, case report

## Abstract

Congenital pulmonary airway malformation (CPAM) is a rare congenital lung lesion that usually remains asymptomatic during the fetal and neonatal period. However, it can occasionally cause prenatal cardiocirculatory failure and fetal hydrops, requiring a thoraco-amniotic shunt (TAS) placement. In other cases, it can also cause symptoms at birth (such as respiratory distress) and may require urgent surgical intervention. Thoracoscopic lobectomy for neonates is rarely reported. Here, we report a case of right macrocystic CPAM causing fetal hydrops at 27 weeks of gestation. The fetus was treated with a TAS placement that successfully resolved the hydrops. At 39 weeks of gestation, a male neonate was born (weight 2,850 g). The TAS spontaneously displaced during delivery, causing an open pneumothorax (PNX), initially treated with a drainage. His condition gradually worsened, requiring ventilatory support. Computed tomography (CT) scan showed different giant cysts in the context of the right lower lobe, left mediastinal shift, and compression of the rest of the lung. An urgent surgical management was required. A thoracoscopic right lower lobectomy was performed at 10 days of life (weight 2,840 g). The postoperative course was uneventful; the child remained totally asymptomatic and showed a good recovery. To the best of our knowledge, this is the first reported case of open iatrogenic PNX following TAS positioning and the second of neonatal thoracoscopic lobectomy in a newborn weighting less than 3 kg. The purpose of this report is to indicate that minimally invasive surgery is feasible, safe, and effective for the resection of CPAM, even in small newborns.

## Introduction

Congenital pulmonary airway malformations (CPAMs) are rare developmental lung anomalies. The majority of CPAMs are antenatally diagnosed and remain asymptomatic after birth. Less frequently, CPAMs can cause acute respiratory distress at birth, requiring mechanical ventilation and urgent surgical intervention. 1 In even rarer cases, CPAMs can complicate prenatally, causing cardiocirculatory failure and leading to life-threatening fetal hydrops, requiring prenatal treatment. One of the recommended fetal interventions consists in a thoraco-amniotic shunt (TAS) placement. 2 Although this procedure can be lifesaving, it can also be complicated by shunt obstruction or displacement. 3

In this report, the authors describe the case of a fetus with a CPAM complicated by hydrops and an acute symptomatic postnatal course, due to a novel complication of TAS needing early treatment.

## Case Report

A male fetus was diagnosed with a right macrocystic CPAM at 27 weeks of gestation during a routine ultrasound (US) scan. A fetal magnetic resonance imaging (MRI) recorded a CPAM volume ratio (CVR) of 2:1 (total size: 52 × 36 × 35 mm) and showed left mediastinal shift and fetal hydrops.

Based on these findings, an amnioreduction and a TAS positioning were performed at 29 weeks of gestation. The procedure led to hydrops resolution and the overall size of the lesion reduced to 22 × 18 mm at 36 weeks of gestation. The remaining gestational course was uneventful.

C-section was performed at 39 weeks of gestation; the birth weight was 2,850 g. During delivery, TAS spontaneously displaced, and a compressive dressing (Tegaderm) was placed on the exit site.

**Fig. 1:**
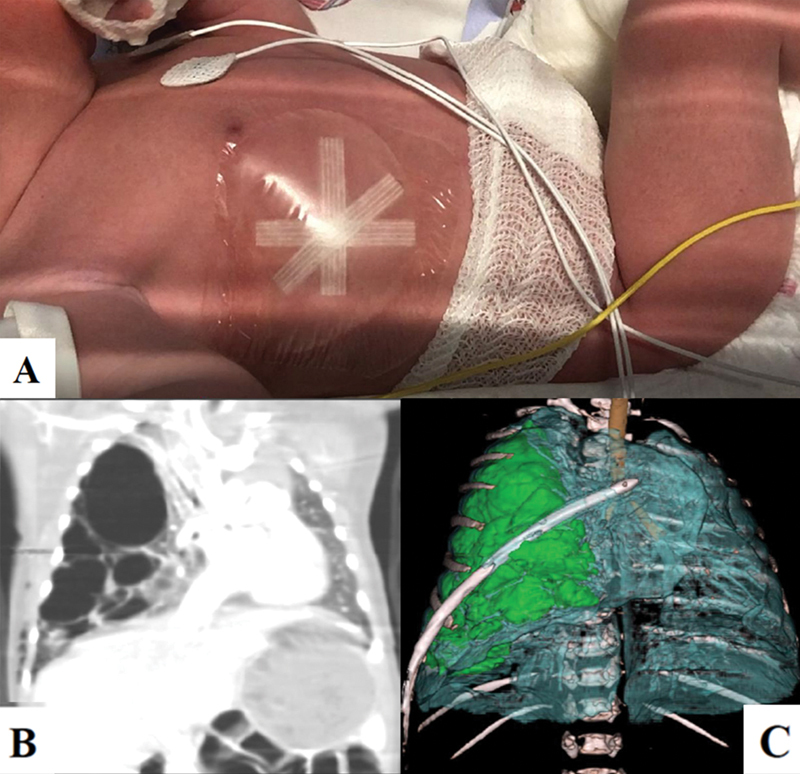
( **A** ) After birth, the thoracic dressing placed at the exit site of the displaced thoraco-amniotic shunt started to inflate, hinting an open connection to the airways. ( **B** ) Computed tomography (CT) scan and ( **C** ) three-dimensional (3D) reconstruction performed at birth: multiple residual cystic lesions ( *highlighted in green* in the 3D reconstruction) and severe left mediastinal shift.

After few hours of clinical stability, the patient developed acute respiratory distress requiring oxygen supply; a chest X-ray showed a right pneumothorax causing left mediastinal shift and the thoracic dressing started to inflate, hinting an open connection to the airways ( Fig. 1A ).

A chest tube was positioned through the site of the previous TAS, with rapid resolution of symptoms and no further need for oxygen therapy. A chest X-ray performed after the procedure showed a correct positioning of the drain, a reduction of the pneumothorax and of the mediastinal shift. A chest computed tomography (CT) scan was then performed, which showed a reduction of the anteromedial cyst (drained by the chest tube) and the persistence of giant posterolateral cysts affecting the right inferior lobe. The middle lobe was difficult to evaluate due to the cysts' compression. The upper right lobe and left lung appeared compressed but free of disease. Three-dimensional (3D) reconstruction allowed us to better define the lesion's features ( Fig. 1B, C ).

The patient remained stable until the 9th day of life, when intubation was required due to severe desaturation (<80%). A chest X-ray detected a hyperinflated right lung causing a severe left mediastinal shift. After intubation, the patient reached a satisfactory clinical state, with normal blood oxygen saturation, heart rate, and arterial pressure, so that surgery was performed the following morning as an urgent procedure but not an emergent one. The patient's conditions were not considered a contraindication to minimally invasive surgery by the anesthesiologist and neonatologist team.

**Fig. 2:**
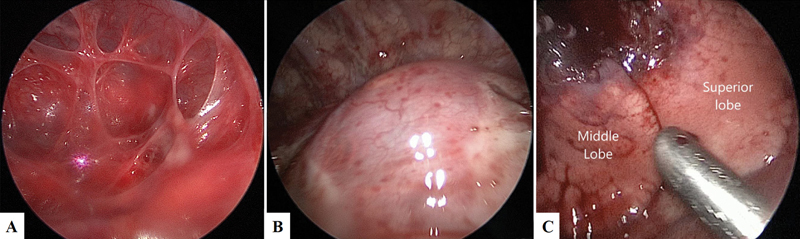
( **A** ) The pleural cavity is occupied by multiple pleural adhesions, probably a consequence of the prenatal thoraco-amniotic shunt (TAS) positioning. ( **B** ) The inferior lobe is completely replaced by multiple giant cysts. ( **C** ) The middle and superior lobes, even if compressed and with atelectasis areas, appear macroscopically unaffected.

On the 10th day of life (weight 2,840 g), a thoracoscopic right lower lobectomy was performed under general anesthesia and tracheal intubation. One 5-mm and two 3-mm ports were used. A 4 mm Hg capnothorax was induced. The pleural cavity appeared to be occupied by multiple pleural adhesions ( Fig. 2A ), probably as a consequence of the prenatal TAS positioning. The inferior lobe was completely replaced by multiple giant cysts ( Fig. 2B ), while the middle and superior lobes appeared macroscopically unaffected ( Fig. 2C ). The bigger cysts were decompressed with the 3-mm JustRight sealer (Bolder Surgical, Louisville, Colorado, United States) to achieve enough intrathoracic working space. Subsequently the fissure was opened, the arteries to the lower lobe were individually isolated and closed with the 3-mm JustRight sealer. The inferior bronchus and vein were then closed with multiple 5-mm Hem-o-lok (Teleflex Inc., Morrisville, North Carolina, United States). An 8Ch (Charrière) chest drain was placed after the procedure. Thoracoscopy with capnothorax was well tolerated by the patient who remained stable throughout the procedure as monitored by traditional and near-infrared spectroscopy (NIRS) monitoring. No signs of acidosis, pathological decrease of cerebral, and splanchnic oxygenation were recorded. Length of surgery was 85 minutes.

The postoperative course was uneventful. The patient was extubated on postoperative (PO) day 4, and the chest drain was removed on PO day 6. Chest X-ray showed re-expansion of the residual right lobes. The pathology report confirmed the diagnosis of type 1 CPAM. After 6 months, a CT scan with 3D reconstruction was performed, showing complete re-expansion of the remaining lobes and lack of disease.

## Discussion

In the antenatal period, CPAM occasionally causes increased intrathoracic pressure, venous return impairment, and consequential fetal hydrops. In these cases, CPAMs are usually treated with the placement of TAS. The risk of hydrops has been related to a CVR > 1.6. 4 Our case had a CVR of 2.1. Meeting all recommended criteria, 5 our patient was treated with amnio-decompression and TAS placement.

Due to its complexity, TAS placement requires highly specialized centers. In our case, it resulted in a safe and effective procedure to resolve hydrops.

As reported in the literature, TAS complications include malposition, obstruction, and displacement into the amniotic or thoracic cavities 6 7 ; only one case of closed tension pneumothorax has been reported. 8 Our case represents the first reported open pneumothorax following TAS displacement, probably caused by a direct communication between a cyst and the external space.

In the absence of previous similar reported experiences, the pneumothorax was drained with a chest tube positioned through the previous TAS insertion site. This conservative management proved to be effective in reducing the mediastinal shift and stabilizing the patient for a few days. However, early surgery was forced by the clinical deterioration of the patient due to expansion of the residual cysts and the urge to preserve the spared lobes from a secondary compression injury.

Thoracoscopic neonatal lobectomy represents an extremely challenging procedure due to the high level of technical skills required, the limited surgical space, and the difficult anesthesiologic management, especially when dealing with symptomatic patients. We chose a thoracoscopic approach building on our previous satisfactory experiences on infantile lobectomies using pediatric-size instruments. 9 10

A multidisciplinary evaluation involving neonatologists, anesthesiologists, and radiologists confirmed the eligibility of the patient for this approach. During surgery, both standard monitoring and NIRS monitoring were performed to promptly identify and manage possible clinical instabilities eventually requiring open conversion. 11

Only few experiences in the literature reported thoracoscopic approach to neonatal CPAM. 12 13

To the best of our knowledge, our experience is the second reported on a neonatal thoracoscopic lobectomy in a newborn weighting less than 3 kg. 14 Our case, however, added a complex prenatal approach with TAS positioning and a challenging surgical management due to the cysts' size and adhesions caused by fetal drainage.

Since there was a suspected involvement of the middle lobe on the preoperative CT scan, thoracoscopy was decisive to assess the actual extent of the disease and decide to limit the resection to the lower lobe.

A multidisciplinary follow-up strategy needs to be planned to monitor the functional and anatomical evolution of the patient. We opted for a thoracic CT scan with 3D reconstruction after 6 months of life because the first CT scan could not exclude the middle lobe involvement. We also thought that thoracic MRI would not have the necessary resolution. Luckily, as the operative image suggested, the middle lobe was free of disease. As further follow-up, we opted for close periodical clinical evaluation.

## Notes

Importance for a Pediatric Surgeon Congenital pulmonary airway malformations can be fatal if associated with fetal hydrops. Combined minimally invasive prenatal intervention and postnatal lobectomy requires a tertiary care center, but can grant survival in such cases.
